# Kindling Kindness for Compassionate Disaster Management

**DOI:** 10.1371/currents.dis.078959ba72f0d133cd2d8fd7c7d9b23d

**Published:** 2015-10-05

**Authors:** Sarb Johal

**Affiliations:** Joint Centre for Disaster Research, Massey University / GNS Science, Wellington, New Zealand

## Abstract

In the health sector, it has become clear that staff who feel better supported deliver better care. Can disaster management learn from this drive to ensure compassionate care to avoid the perils of burnout and empathy exhaustion?

## Perspective

In recent years, evidence has emerged that disaster management places significant burdens on those tasked with carrying out planning, response and recovery functions, especially where human contact is involved^1^^,^ 2 ^,^ 3. Research has identified that rescue and recovery workers engaged in disaster relief are at increased risk of developing mental health problems such as post-traumatic stress disorder, depression and anxiety^4^. They are also at increased risk of empathy exhaustion, burnout, compassion fatigue, and vicarious traumatisation^5^. In addition to this, an increasing number of hereto thought of low-frequency-high-impact events has placed further pressure on resource allocation issues, as well as calls and new frameworks proposed for reform about how disasters are managed in the context of sustainable economies in the face of climate change at local, regional, and global levels (e.g. Sendai Framework for Disaster Risk Reduction 2015-2030^6^).

The challenge of approaching disaster management across all sectors of society and the economy while paying attention to the human impacts of identified risks and as well as the impacts of preferred mitigation solutions is a mammoth task of scale and coordination. Implementing a framework to meet these challenges is an even bigger task.

In the health sector, patients problems are becoming increasingly complex and the care provided for them more and more fragmented. Efficiency savings, greater population disease burden, and constant pressure for cost savings place increasing pressure on resources, along with strain on relationships between staff members, and staff and the patients they care for. We know that all staff members in the healthcare professions are affected by the emotional demands of caring for patients. Whether this shows itself in increased rates of work related anxiety and depression absentees, or staff burnout, both clinical and non-clinical staff are at risk. In such circumstances, it has been noticed that care can become rapidly depersonalised with sometimes catastrophic consequences^7^.

One possible solution to this sense of depersonalisation and burnout has emerged from the experiences and writing of Ken Schwartz. During a harrowing 10-month ordeal before Schwartz died of advanced lung cancer, he recorded his reflection that what mattered most during an illness is the human connection between patients and their caregivers. In his book, ‘A Patient’s Story, he encouraged healthcare professionals to stay person-centred: “…the smallest acts of kindness”, he argues, make “the unbearable bearable”^8^.

Schwartz recognised that one way of supporting staff through the brutality of their everyday experience was to give them the space to reflect and talk - a space to be able to tell stories about things that happened to them during their work. The mission of the Schwartz Centre for Compassionate Healthcare, established after Ken Schwartz’s death, was to promote compassionate care so that patients and their caregivers relate to one another that offers "hope to the patient, support to caregivers, and sustenance to the healing process”^8^.

Schwartz Centre ‘Rounds’ are a multidisciplinary forum designed for staff together once a month to discuss and reflect on the non-clinical aspect of caring for patients, that is the emotional and social challenges associated with their jobs. Schwartz Rounds have been successfully running in hospitals in the USA for over 17 years, and have also been used to good effect in the UK^9^. The general format of Rounds is as follows: a pre-selected panel spend 10-15 minutes presenting a case story and describing their role, the issues the case raised for them, and how this made them feel. It is critical not to be diverted into the technical aspects of the case, as per a usual hospital Case Round, but to remain with the how the case made them feel. Under the guidance of a skilled facilitator, discussion then opens up to the larger group of participants for the remainder of the hour long meeting, asking questions to encourage sharing of experiences and to reflect on the challenges of care. Rounds are designed to be a safe, confidential environment that are not designed to focus on problem solving, but to instead to consider the implications of the case for staff. Rounds are generally held over lunch, with food provided for staff (which seems to be a critical part of the Rounds’ recipe for success).

The underlying premise for Rounds is that compassion shown by staff can make all the difference to a patient’s experience of care. In order to provide care with compassion, however, staff must in turn feel supported in their work. What the Schwartz Centre promotes is no ordinary ‘debriefing’ experience. In broad terms, staff are unprepared for and unaccustomed to reflective practice. Staff are rarely encouraged to stop to consider how their work feels, or what it means to do the work they do, for example, the ethical dilemmas, the existential issues of dealing with life and death, or the day-to-day stresses and rewards.

Schwartz Rounds are not intended to produce actionable outputs at the end of the process. Instead, their value lies in the process of both recounting the story from an overtly subjective point of view, and the act of listening and responding. These spaces are intended as times to be able to recognise and discuss the processes that healthcare workers find themselves deeply involved in. They provide an opportunity to share narratives with one another, and provide an experience through which to socialise us to be able to do so.

Staff who have participated in rounds report that they feel better supported in their patient care, and their levels of stress and isolation have been shown to decline. Furthermore, it was found that the more rounds attended, the greater the positive impact on staff^10^ and it seems that the very act of attending Rounds regularly focuses staff attention on the need for compassion. A separate study of regular Rounds attendees concluded that compassionate caring requires “a lifetime of continuous support”^11^.

In the field of disaster management, Schwartz Rounds have been used with some success. In July 2014, the Schwartz Centre published a White Paper describing how the protocol was used to help caregivers to collectively process the complex and challenging feelings and emotions that may arise when caring for the injured and dying after a traumatic event - in this case, the Boston Marathon bombing^12^. It is notable that one of the facilitators observed how she was struck by the fear that people were facing about attending the race one year later, indicating that they were not just processing the past but also facing the fear that something dangerous could also happen in the future^12^.

The World Conference on Disaster Risk Reduction in Sendai^6^ recently issued a 15-year action plan urging countries forward on several fronts. Five of the seven targets identified are particularly relevant for health. Moreover, this Framework is only one of four global level deals to be finalised this year, the others being on sustainable development and climate change, as well as the first World Humanitarian Summit in 2016. More and more targets and processes continue to emerge that place ever increasing burdens on disaster management staff, in all parts of the policy and practice arena

There is a risk that in focusing on the delivery of actions related to these international frameworks that disaster management becomes depersonalised, and becomes disconnected in a critical way from its core goals of reducing risks and impacts of disasters while improving lives and livelihoods. In conjunction with these developments, and increasing reliance and focus on using ‘Big Data’ to unlock some of the public health challenges of the modern world can lead to the use of cognitive heuristics that can lead to both blindness to scale and empathy loss. Though there is little doubt that a more strategic and purposeful interrogation of complex, large datasets may result in fresh insights to deliver the core goals of DRR, this is but one of many tools available to disaster and health managers. The associated risk is that large numbers and datasets can be dehumanising, and disaster management and health professionals need to be sensitised to this^13^.

The ethos and protocols offered by Schwartz Rounds offers an opportunity to reduce an increased risk of dehumanisation and empathy loss that a focus on global scale frameworks, or international/national/regional datasets might bring. In healthcare settings it has not been unusual for a false dichotomy to be set-up: that once must choose between compassionate or competent care, assuming that you cannot have both. The evidence indicates that this is untrue^14^. Organisations that focus on delivering compassionate care benefit from lower staff turnover, higher retention, recruitment of more highly qualified staff, and better health outcomes. Moreover, caregivers who are able to express compassion for patients, families, and each other experience higher job satisfaction, less stress and a greater sense of teamwork. A similar set of processes supported by careful facilitation that enable disaster managers and their interdisciplinary colleagues to regularly discuss the social and emotional dimensions of their work in an open and honest manner may help to deliver similar benefits in disaster management settings. Staff can be provided with an opportunity to share their experiences, thoughts and feelings on thought-proving subjects drawn from real-life disaster response and recovery cases. The critical premise is that staff are better able to make personal connections with their colleagues and those they are trying to assist when they have greater insight into their own responses and feelings. In this way, Schwartz Rounds or similar processes can decrease feelings of stress and isolation, and increase openness to giving and receiving support.

A focus on the impact of the human scale and impact of working in disaster management, whether in a health context or more broadly, can help to increase the sense that staff feel supported in their work, and can still be in touch with their empathic concern when working in difficult contents - from active disaster response to working to deliver actions determined by international agreements. In this way, we can help and support our most valued resource - our skilled workforce - to deliver effective, competent, and compassionate disaster management.

He aha te mea nui o te ao

What is the most important thing in the world?

He tangata, he tangata, he tangata

It is the people, it is the people, it is the people.

Māori proverb
